# Logarithms—A Neglected Help

**Published:** 1916-05

**Authors:** 


### Logarithms—A Neglected Help.

If the physician engages in quantitative work of any kind, such as the calculation of calories from a diet list, the actual amount of uric acid, urea etc., in urine, the the total fat in faeces estimated from a small amount in a sample which is not usually an aliquot part of the entire discharge, the combining quantities of fat and alkali in soap, or if he has to translate pounds and ounces into grams, he has many problems in multiplication, division and proportion. As a business man, he also has similar problems to consider, as the calculation of a tax from rate of so many dollars and cents per thousand; interest for odd fractions of a year; area and capacity of rooms, barns etc. Recently, we have been interested in estimating the practical investment value of life insurance as compared with compound interest at a bank. On inquiry of several acquaintances in the banking business, we learned that no tables were available showing the aggregate amount of a given sum at a certain rate of interest, compounded at stated intervals for a term of years. We readily found, however, in an advanced algebra, the following formula: A (aggregate amount)=(1 plus r) $^{n}$, p being the principal, r the interest, n the number of years, if the compounding is done annually. If interest is compounded semi-annually, the formula becomes: $A=P\ (1+\frac{1}{2}r)^{2n}$.

This looks simple enough but when one raises 1.02 to the 40th, 39th and successive diminishing powers, and multiplies this by dollars and cents in two figures each, the amount of arithmetic work is appalling.

We would scarcely go so far as to advise the physician to use a table of logarithms in calculating the totals in a twenty-dose metric prescription or in a 24-dose apothecary's prescription but for any multiplication or division requiring the use of three or four figures in each member, especially when a mass of similar problems are presented, much time may be saved by this method.

We need not here enter into the theory of logarithms but would merely advise a brushing up by reference to the old high-school geometry and trigonometry or to an advanced arithmetic or algebra.

Engineers have a wonderful little instrument called the slide in which a scale of equal parts corresponds to another scale which increases proportionately to the logarithms of the numbers of the former. By this instrument, especially in its more elaboate forms, pretty nearly every problem involving multiplication and division can be performed mechanically, with a chance of error of about 1: 1000. The rate of flow of streams, the capacity of all sorts of containers, the determination of cicumferences and areas from diameters, the conversion of one set of units into another and many much more astonishing problems in astronomy, engineering and other applied mathematics, can be gotten by this instrument. Unfortunately, to secure sufficient accuracy, a somewhat expensive instrument is necessary and considerable skill is required to work it accurately. In fact, a description of its many uses makes up a good sized book.

